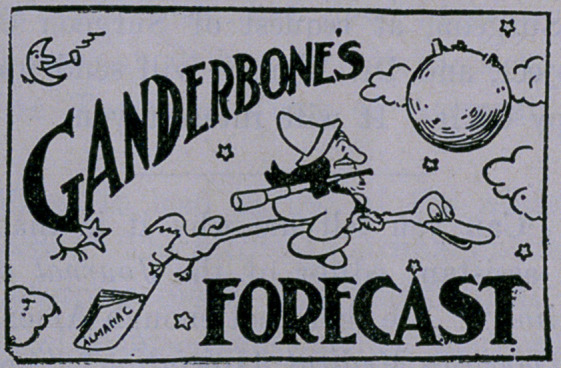# Abstracts and Selections

**Published:** 1908-12

**Authors:** 


### Abstracts and Selections.

**For December.**

(Copyright 1908 by C. H. Rieth.)

Save, brothers, save with care,
A nickel here and a nickel there—
A two-bit piece will fill the tum
Of some small celebrant with gum,
Nigger-toes, peanuts, bum-bum,
Apples, raisins, lemon drops,
Popcorn, suckers, caramels,
Filberts, taffy, butterscotch,
Walnuts, figs and angel cake—
Save for the Christmas bellyache.

Save, brothers, save your dough,
Save for the stockings in a row—
A four-bit piece will buy a sled,
A pair of boots upholstered red,

A doll with skullgrass on her head,
A Teddy bear, a horn, a drum,
An airgun, jack-knife, pair of skates,
Magic lantern, doll house, game,
A box of soldiers made of tin—
Save for the Christmas morning din.

Save, brothers, save for keeps,
Save for the night nobody sleeps—
A six-bit piece will buy a book,
A piece of cat fur for the cook,
A picture for the empty nook,
A box of holiday cigars,
A hatpin, slippers, pair of mitts,
A dozen handkerchiefs, a shirt,
A piece of neckwear new and strange—
Save for the annual exchange.

December was the tenth month in the old Roman year, and gets its name from the Latin decem (10). But what with forest fires in the middle of autumn, the Roman fire department had no time to put out Christmas trees, and when Numa's own palace burned he rearranged the calendar and moved Christmas along to a time when the firemen had nothing else on.

The frisky calf will sniff the morn and merrily cavort, and the frost will nip him where his scant upholstering is short. The boys will flock to Sunday school with fine religious fire, the hired man will hang around unworthy of his hire, the warning goose will hurry south on frantic wings a-rustle, with winter urging him along where Cora wore the bustle.

The melancholy days will come, and Boreas will roar; the wolf will thrust his muzzle through the keyhole in the door. He'll whiff the scent of bacon bought at 30 cents a pound, and plain potatoes by the box with tissue wrapped around; and every time we chase him off and bid him to his den, the trusts will pump his stomach out and sic him on again.

The water pipes will all explode
And give the house a jar;
The plumber's chauffeur will get out
The throbbing racing car;
But while relief is on the way
To plug the gushing spout,
The car will have a hemorrhage
And blow its innards out.

The unemancipated wife will quit her cozy bed and build the fire the while her lord pretends that he is dead. She'll wrap herself up in her hair and shiver in the dawn, and chop the kindling, hustle coal and turn the damper on; and while she freezes till she turns the hue of a persimmon, the scientists will wonder why the men outlive the women.

Milady's winter suit will cost
  A fifty-dollar note,
But man will face the blizzard in
  The same old overcoat.
The cook will gasoline the fire,
  And the choir, in sweet accord,
Will sing on Sunday at the house,
  "O, I am coming, Lord!"

On the 21st the sun will enter Capricorn, and the winter solstice will occur. This will cause trusts to spawn, and the yule tide will come in. Solstice is from sol (the sun) and sistere (to stand). Thus, having touched its turning point south of the equator, the sun temporarily stands still. This was where Joshua held it up, from which we have our word josh. Anybody can do it.

At any rate, the 21st will be the shortest day of the year, and we shall pass under the influence of Capricorn, the Goat. This is the tenth sign of the zodiac. It was represented on the ancient monuments as an old man with the body of a goat, which signifies that during the Christmas season father is the goat.

The Christmas shoppers' Marathon
  Will happen as of yore,
And the little savings bank will chase
  The present round the store.
The unleashed dollar will pursue
  The frightened Teddy bear,
The crowd will trample on the clerks
  And pull each other's hair;
The young and old will pull and push,
  And mill around and butt,
And Santa Claus in terror will
  Take to the tall uncut.

And then the fateful Christmas Eve will come with wintry weather, and Morgan will hang up his shirt with both tails pinned together; and anything by way of luck that manages to miss its yawning neck we're welcome to, O joy and double bliss!

The happy kids will rise at morn
With rapture at the bat
And get down twenty-seven steps
With turn in nothing flat.
The tickled heart of youth will dance
And sing its wondrous luck,
The cautious boy will climb the flue
To see if any stuck,
The house will oscillate with joy,
The breakfast will be late,
And old John D. will get the earth
For thirty winters straight.

Mr. Roosevelt will give a big game dinner at the White House Christmas day. All the big lion hunters of the world will attend, and there will be a roaring contest with favors. The password for this month is supplied by the Kaiser William. Mum is the word. The moon will be full on the 7th, and there will be bad weather around 26 Broadway every day but Sunday.

The mistletoe will tempt the maid,
Beneath the chandelier,
The loving swain will halt and start
Betwixt his love and fear,
And then dash in with beating heart
And chew her blushing ear.

The birthstone for December is the onyx. This is because children are onyxpected at a time when the stork has to make his deliveries in a fireless cooker.

Then Leap Year will have run its course
With little to its merit,
And the spinster who has missed her chance
Will have to buy a parrot.

---

### Pyorrhea Alveloaris.

*Editor Texas Medical Journal:*

In the Texas Medical Journal for October I noted an interesting paper on Pyorrhea Alveolaris, by Dr. Julian Smith. The keynote of this excellent paper is given in the words: "The systematic cause is autointoxication." As this malady has been for many ears the *bete noir* of the dental profession, it is well worthy of extended consideration.

The connection of pyorrhea with autointoxication and the treatment deduced from that discovery emanated from the group of men who are advocating the alkaloids. The late Dr. E. L. Clifford, a dentist of note, a keen observer, who had highly distinguished himself in his profession, become interested in the work going on in the alkaloidal circles, and especially in the doctrine we have so persistently urged, of the cardinal importance of the "clean out, clean up and keep clean" principle. The symptoms of autotoxemia from fecal retention and absorption struck him as being essentially those he had observed in his cases of pyorrhea. He proceeded to apply to the latter the methods of treatment which had proved effective in our hands, and the result was so satisfactory that he felt he had made a discovery of the first importance. So impressed was he with the value of this to the dental profession that he even considered the question of devoting himself to the duty of disseminating a knowledge of it among his brethren; and this might have been undertaken but for his sudden death by accident.

Dr. Clifford brought this matter to the attention of Prof. E. S. Talbot, who at once recognized its vast importance and took it up in his thorough methodical way. He spent an entire year in experiment and observation before he permitted himself to give an opinion upon the merits of the question; but, when he did, it was in no uncertain manner. When Professor Talbot speaks thus plainly, all who know him are aware that he is satisfied himself, and has the testimony to prove every assertion he makes.

As elaborated by these gentlemen, the internal treatment of pyorrhea alveolaris is simple and comprehensible. The diet must be regulated; the bowels emptied and kept regular; the suppuration, if any exists, is to be stopped by the administration of the sulphide of lime or of arsenic, and beyond that the local treatment is strictly dental. Professor Talbot has devised a most ingenious apparatus for measuring the relative acidity of the urine and detecting the presence of indican. He places great importance on these points, and finds attention to them gives satisfactory results.

The phsyician who is familiar with this doctrine, which I have preached so persistently for years, will have no difficulty when he recognizes in pyorrhea alveolaris one more evidence of fecal autotoxemia, to be placed with the other familiar evidence of that condition—bad breath, anorexia, pruritus, sluggishness, pimply skin, muddy conjunctiva, etc. The morning saline laxative does away with the source of these, and many anomalous symptoms, which commonly go under the name of "biliousness," "bad blood," of late are attributed to uric acid, and I fear too often ascribed to malaria. The man who begins to study the bowels and the effects of retained fecal matter clinically, will not long accuse me of overestimating their importance.

W. C. ABBOTT.

---

### Army Medical Corps Examinations.

---

*Texas Medical Journal:*

The Surgeon General of the Army announces that the first of the preliminary examinations for the appointment of first lieutenants in the army medical corps for the year 1909 will be held on January 11, 1909, at points to be hereafter designated.

Full information concerning the examination can be procured upon application to the Surgeon General, U. S. Army, Washington, D. C. The essential requirements to securing an invitation are that the applicant shall be a citizen of the United States, shall be between 22 and 30 years of age, a graduate of a medical school legally authorized to confer the degree of doctor of medicine, shall be of good moral character and habits, and shall have had at least one year's hospital training or its equivalent in practice. The examinations will be held concurrently throughout the country at points where boards can be convened. Due consideration will be given to localities from which applications are received, in order to lessen the traveling expenses of applicants as much as possible.

The examination in subjects of general education (mathematics, geography, history, general literature, and Latin) may be omitted in the case of applicants holding diplomas from reputable literary or scientific colleges, normal schools or high schools, or graduates of medical schools which require an entrance examination satisfactory to the faculty of the Army Medical School.

In order to perfect all necessary arrangements for the examination, applictions must be complete and in possession of the Adjutant General on or before December 10, 1908. Early attention is therefore enjoined upon all intending applicants. There are at present fifty-seven vacancies in the Medical Corps of the Army.

### Anti-Tuberculosis.

The following resolutions were adopted by the Southwest Medical Association at the Kansas City meeting:

WHEREAS, The tendency of physicians and charitable organizations over the country is even now to send advanced, indigent consumptives from their homes to climatic resorts, notably parts of Texas, Colorado and the Southwest; and

WHEREAS, The consensus of opinion among the best authorities is that climate alone can not cure tuberculosis; and

WHEREAS, Boarding houses and hotels in many resorts no longer open their doors to this class of people, thereby depriving them of any chance of securing proper accommodation; and

WHEREAS, The sanitariums and eleemosynary institutions of the Southwest are already overburdened with such cases, and the people are called upon to do double duty in that they must take care of others besides their own consumptives; therefore, be it

*Resolved,* That all States and Territories throughout the country, and all physicians and charitable organizations be urged to discourage the aimless drifting of the average consumptive, and that all advanced consumptives be kept within the confines of their own city, county or State, and that the Legislatures of the several States be urged to pass such laws as will insure the building and maintenance of sanitariums for curable cases and hospitals for the advanced and incurable cases.

Upon motion, duly seconded and carried, the above motions were unanimously adopted. The secretary was also authorized to notify the secretary of each State Association of this action.

### State Medical Association.

STATE MEDICAL ASSOCIATION OF TEXAS.
OFFICE OF THE PRESIDENT.

HEARNE, TEXAS, October 23, 1908.

*To Chairmen and Secretaries of Scientific Sections, State Medical Association.*

GENTLEMEN: Feeling that each of you join me in an earnest desire to make the next annual meeting of the State Association one of the most interesting as well as profitable in the history of the organization, and realizing that within the next two months many of the most important district and county meetings will be held, I am calling your especial attention to the matter of beginning work upon the program for the next meeting.

As I understand the by-laws of the Association, with the exception of special papers, you must secure your program from those papers which have been presented to the various district and county societies during the year.

Will you not therefore begin as early as possible to make inquiry of those who have written papers of interest, as well as to request of such men, as you think will do so, to prepare a paper upon some subject suitable to your section?

In the arrangement of the program, I shall endeavor to so place the sectional meetings as to conflict as little as possible, and I trust that we may succeed in making the entire time of much interest and value to those who attend the sessions, as well as give due consideration to those who take the pains to favor us with their contributions.

I have the promise of two or three prominent physicians out of the State to visit us at our meeting in Galveston, and make addresses which I am sure will add much to the value of the meeting.

If any chairman or secretary has in mind any one not a member of the Association to whom he would like to extend an invitation to be with us, kindly do so and upon notice I will add my request.

Those who are members of the Association who have papers which they would like to present will please communicate to the chairman of such section as title might indicate.

Trusting that the chairmen and secretaries will get in touch with one another as early as convenient to begin the work, and assuring you of my confidence that your sectional work will reflect credit both upon you and the Association, I am

Very truly yours,

H. W. Cummings,
President.

Section on State Medicine—Dr. F. E. Daniel, Austin, Chairman; Dr. L. B. Bibb, Austin, Secretary.

Those who wish to read papers in the Section on State Medicine are requested to send title to the secretary as soon as possible.

L. B. Bibb, M. D., Secretary,
Austin, Texas.

### The Saloon.

In the *Americal Journal of Clinical Medicine,* Dr. C. W. Woods, of Broadus, Texas, has a strong article on "The Saloon." We extract the following, and add our earnest endorsement of every word of it:

"What is a saloon? It is a parasite. It takes money from people and gives them nothing of value in return. Saloon keepers are not producers. They live off the people, and not only do this, but in return for their money make them drunkards and criminals, fill our jails, penitentiaries and insane asylums, make widows and orphans and worse still destroy virtue and both soul and body. * * * This government has no moral right to license an evil. This government licenses men to sell poison that befuddles men's brains, makes them temporarily insane, then punishes them for the crime they commit while under the influence of the hell-born stuff. The saloon keeper should be punished by confinement in the penitentiary for every felony committed by the drunkard, and the drunkard confined on the convict farm until he reforms, or better yet, make him work on the public roads of the country, and the government pay him a reasonable sum for his labor and give it directly to his family.

It is coming. The time is not far distant when the liquor traffic will be banished from the country. That and that alone will solve the temperance problem.

With a majority of doctors, ninety-nine of a hundred of the true preachers, a large percentage of congressmen and legisators, all true, pure women, and 50 per cent of American-born voters against the liquor traffic, one can see that the day is not far distant when it will hide its venomous head, leave 'the land of the free and the home of the brave,' and be forced from the country. God speed the day!"

### New Use for an Old Drug.

The *Medical Council* for October notes the fact that the newest and most interesting feature in therapeutics today is the use of magnesium sulphate in the topical treatment of acute and subacute inflammations. The results are such that doctors quickly become enthusiastic in its utilization. Large quantities of Epsom salts in saturated solution are now being employed and the routine work of many hospital departments and clinics is completely

For Seasickness.—In the *Journal de médecine de Paris* Schepelman is credited with the following remedy for seasickness:

℞ Cocaine hydrochloride.....................gr. iii
Tincture of iodine.......................gtt. xxx
Distilled water ............................ʒv

M. Sig.: One tablespoonful for a dose, to be repeated two or three times, as needed.

---

A Tonic for Tuberculous Patients.—The following tonic mixture is prescribed in cachets by Sergent (*Journal de médecine de Paris*):

℞ Calcium carbonate.........................gr. 1
Tricalcium phosphate .......................ʒiss
Calcined magnesia .......................gr. xviii
Sodium chloride..........................gr. xviii

M. ft. et div. in cachet No. xii.
Sig.: One cachet three or four times daily with meals.

## Figures and Tables

**Figure f1:**